# From naked eye to dermoscopy: Closing a diagnostic gap in South African primary care

**DOI:** 10.4102/safp.v68i1.6367

**Published:** 2026-08-28

**Authors:** Magrietha Swart

**Affiliations:** 1 Dr AP Swart Inc., Molteno Anaesthetics, Cape Town, South Africa

**Keywords:** primary healthcare, dermoscopy, general practitioners, medical education, South Africa

## Abstract

Skin pathology is among the commonest reasons for South African primary care consultations, yet the dermatology workforce is small and unevenly distributed. Only 264 dermatologists serve a population of over 60 million, at 20.1 per million in the private sector and 1.2 per million in the public sector. Most patients with skin-related concerns usually present to general practitioners or family physicians first, who largely work with naked-eye examination alone. Dermoscopy is an evidence-based tool that significantly improves the accuracy of skin lesion assessment and is becoming routinely used in primary care in countries such as the United Kingdom and Australia.

**Contribution:**

This article argues that dermoscopy should be reconsidered as a possible primary care skill in South Africa, keeping in mind that implementation is dependent on necessary equipment and training.

## Introduction

Skin pathology complaints remain one of the commonest presenting issues in South African primary care, yet dermoscopy is an underutilised tool in South Africa and generally only something that dermatologists use. General practitioners (GPs) and family physicians rely on naked-eye observations and patient history, and with only 264 dermatologists registered with the Health Professions Council of South Africa at the most recent national audit^1^ – distributed at 20.1 per million in the private sector versus 1.2 per million in the public sector – waiting times can be long. Primary care may need to expand into using tools like dermoscopy: a practical, evidence-based tool that could improve diagnostic accuracy and referral decisions.

South Africa has among the highest ultraviolet indices in the world, and skin cancers remain one of the most commonly occurring malignancies. Although there is a clear predilection for lighter-skinned patients, skin cancer does affect all population groups, and in darker-skinned patients, lesions often present at advanced stages and at atypical sites like soles, nails and mucosa.^2^ These pressures are unlikely to ease soon: global estimates are that the annual number of new melanoma cases will rise by more than 50% between 2020 and 2040, with deaths rising by roughly two-thirds over the same period^3^, and South African registry data show that melanoma incidence among the white population has been rising since the 1990s.^4^ Although countries’ healthcare systems differ significantly, the implication is that South Africa would have a similar burden. Dermatologists are scarce overall, with some provinces particularly underserved. Limpopo, the fifth most populous province, has only four dermatologists serving a 6 million population^1^ – the majority of skin concerns will likely present to primary care providers first. It is therefore essential that primary care providers are able to effectively diagnose skin cancers at first presentation. To avoid misdiagnoses and inappropriate referrals, dermoscopy becomes an effective tool which can significantly improve diagnostic accuracy.^5^ This is especially helpful in cases where patients present with a multitude of skin lesions that are far too plentiful to biopsy.

## Dermoscopy internationally

Dermoscopy has been a mainstay of everyday dermatology practices since the 2000s, and with the adjuvant help of artificial intelligence (AI)-based pattern recognition, it is likely to be even more effective in future. With appropriate training, primary care physicians can readily use dermoscopy not only to diagnose suspicious lesions in need of biopsy,^6^ but also to support the diagnosis of other inflammatory and infectious dermatoses, like scabies and tinea. The result is a measurable reduction in unnecessary biopsies: once dermoscopy entered into routine practice elsewhere, the benign-to-malignant ratio of excised pigmented lesions fell from 18:1 to 4:1 with no comparable improvement in non-users,^7^ and primary care practitioners who used dermoscopy reported greater diagnostic accuracy and confidence when assessing skin lesions.^6^

Internationally, more primary care doctors in countries like the UK and Australia are incorporating dermoscopy into their practices, with some health systems introducing dermoscopy training at medical school level to expand the diagnostic scope of all newly qualified physicians. In the UK, GPs with a Special Interest in dermatology use it routinely, supporting the National Institute for Health and Care Excellence guidelines for the assessment of suspicious skin lesions^8^ and in Australia 98.9% of GPs surveyed report using dermoscopy at least occasionally^9^ in practice. This is supported by formal postgraduate dermoscopy training programmes.

## Prospects for dermoscopy in South African primary care

South Africa’s heterogeneous patient population, with its wide ethnic and clinical spectrum, makes it an especially valuable setting to adopt an essential skill like dermoscopy. It is not only effective in the diagnosis of skin cancers, but also in the infectious and inflammatory diseases of the skin. Unfortunately, the barriers familiar to any middle-income country exist. The undergraduate training in dermatology is sparse, with short rotations throughout the 5–6-year curriculum. Some GPs find the need to complete postgraduate courses or even international diplomas in dermatology to gain adequate knowledge of the speciality. The structural barrier to teaching dermoscopy is therefore considerable.

Cost is another barrier. A clinically usable dermatoscope can range from R10 000 to well over R25 000 for a higher-end model with polarised and non-polarised modes and digital connectivity. This is not a trivial cost for a private GP, and it could be an outright obstacle in the public sector, although many university hospitals have dermatoscopes for their registrar and consultant use. On a per consultation basis, however, the cost is modest. A single melanoma caught earlier or a few unnecessary excisions avoided easily justifies the outlay.^10^

The lack of education in dermatology and dermoscopy substantially skews referral patterns: benign conditions are over-referred to specialist clinics, while skin cancers can reach a dermatologist months, or even years, after their initial presentation. A South African cohort study at Tygerberg Hospital documented a mean total delay of 11.1 months between observed change in a melanoma lesion and appropriate therapy; while most of this was patient-related, professional delay occurred in 12.4% of cases and added, on average, a further 1.3 months to time-to-treatment.^11^ The result is a system that simultaneously underdiagnoses serious skin cancer and over-refers conditions that could have been confidently managed in primary care with dermoscopy skill.

How can this be addressed? More training at the undergraduate level would be beneficial to introduce the dermoscopic features alongside the teaching of skin cancers and skin disease to students, so that they can diagnose and biopsy correct lesions. Greater availability for dermatology postgraduate courses and training, including a possible Colleges of Medicine of South Africa (CMSA) accredited diploma and more Continuous Professional Development (CPD) accredited courses on the topic, would also help. The University of Cape Town currently has a 6-week dermatology course for GPs to reach more primary care providers. There is local evidence that this kind of upskilling works: Makaula et al. showed that basic dermatology education improved diagnostic knowledge among doctors and nurses across three primary care districts in KwaZulu-Natal.^12^ The encouraging finding for dermoscopy specifically is that the learning seems to be easily retained. Even brief courses produce measurable, durable gains: a 1-day workshop for GPs in Sweden raised median diagnostic test scores from 13 to 20 out of 30, an improvement still present at 6 months,^13^ and a randomised controlled trial of a structured training programme demonstrated sustained improvements in lesion triage among primary care doctors.^6^ Most importantly, these gains translate to real-world improvements: a 2024 study found that primary care providers who continued to use dermoscopy in clinical practice achieved a 31% improvement in diagnostic accuracy at 1 year compared with their own pre-training baseline.^14^ Such training would also encourage GPs or institutions to acquire dermatoscopes, once the utility and ease become apparent. Thereafter, utilising quality assurance systems and refresher courses, the gains made can be maintained and evaluated. In this way, the competence of GPs can be assessed and improved, as successful usage of dermoscopy in primary care depends on both the availability of equipment and sufficient and ongoing training of staff.

South African primary care has not been passive in the face of this gap. Many GPs have utilised informal WhatsApp dermatology groups, in which GPs share clinical photographs with consultant dermatologists who volunteer their time, to manage dermatological queries. The Vula app (a widely used tele-referral platform developed by a clinician) has been particularly helpful in the field of dermatology in South Africa. A recent analysis of more than 5800 dermatology referrals at Tygerberg Hospital found that 55% were resolved remotely without an in-person visit and 83.5% included specialist case-based teaching for the referring clinician.^15^ These channels are genuinely valuable but reactive; they help with the lesion that has already been recognised as needing more than a naked-eye assessment. They do not substitute for the diagnostic capability at the point of first contact, which is what proper dermoscopy training would provide. Unfortunately, the implementation of dermoscopy in South African primary care has been very limited, and as a consequence, little data are available. Further local research would be useful to compare with the international outcomes.

## Conclusion

In a healthcare system defined by resource constraints, tools that improve diagnostic accuracy without substantially increasing cost are rare. Dermoscopy is one such tool. Its implementation would require equipment, training, evaluation and ongoing quality assurance in the primary care setting, but its integration into South African primary care represents a practical and achievable step towards improving dermatological care at the frontline.
